# Exploring the relationship between remnant cholesterol and diabetic kidney disease in Chinese type 2 diabetes patients

**DOI:** 10.3389/fnut.2026.1793007

**Published:** 2026-03-30

**Authors:** Yanli Li, Zhuoqi Xu, Dong Xiao, Xiaoyue Yun, Chenfan Liu, Qiwen Xiao, Chao Li, Yin Liang, Dandan Chen, Tao Du, Wangen Li, Ai Luo, Zhuoqing Hu

**Affiliations:** 1 Department of Endocrinology, The Second Affiliated Hospital, Guangzhou Medical University, Guangzhou, China; 2 Guangzhou Medical University, Guangzhou, China; 3 Department of Clinical Laboratory, The Second Affiliated Hospital, Guangzhou Medical University, Guangzhou, China; 4 Guangdong Medical University, Zhanjiang, China; 5 Department of Endocrinology, The Fourth Affiliated Hospital, Guangzhou Medical University, Guangzhou, China

**Keywords:** Chinese, diabetic kidney disease, dyslipidemia, remnant cholesterol, type 2 diabetes mellitus

## Abstract

**Background:**

Remnant cholesterol (RC) has been established as an independent risk factor for atherosclerotic cardiovascular disease. While the association between RC and diabetic kidney disease (DKD) remains unclear.

**Methods:**

This cross-sectional study included 1,893 patients with T2D hospitalized across multiple centers from 2019 to 2024. The correlation of RC and DKD was analyzed by multiple logistic regression and restricted cubic spline (RCS) models. The subgroup analysis was to assess the stability of the correlation between RC and DKD.

**Results:**

The participants comprise 1,340 without DKD and 553 with DKD. RC was significantly higher in DKD patients compared non-DKD patients (*P* = 0.012). In multiple logistic regression analysis, the results revealed a 43% increased DKD risk per 0.1 mmol/L RC increment (adjusted OR = 1.43, 95% CI = 1.10–1.86). Additionally, when analyzed as quartiles, participants in the highest RC quartile (Q4: >0.700 mmol/L) demonstrated 1.77-fold higher DKD risk compared to the lowest quartile (95% CI = 1.28–2.45, *P* = 0.001), with significant linear trend across quartiles (*P* < 0.001). Furthermore, RCS model demonstrated a biphasic relationship: risk increased linearly with RC levels below 0.96 mmol/L (β = 2.25 per 0.1 mmol/L, *P* = 0.001), transitioning to a plateau (β = 0.86, *P* = 0.588) with RC levels exceeded 0.96, suggesting lipid-mediated renal injury pathways may reach saturation. Subgroup analyses confirmed stability across demographic or clinical strata (all *P* > 0.05).

**Conclusions:**

Our study establishes RC as an independent DKD biomarker in Chinese T2D patient, suggesting its dual utility as a pathophysiological indicator and preventive therapeutic target.

## Introduction

The global prevalence of T2D has risen dramatically, making it one of the most pressing public health challenges. Among the microvascular complications of T2D, diabetic kidney disease (DKD) affects nearly 40% of patients, representing a significant cause of chronic kidney disease (CKD) and the leading contributor to end-stage renal disease (ESRD) (1). The progression to ESRD necessitates renal replacement therapy, which severely compromises patients' quality of life and imposes a substantial economic burden on healthcare systems. Despite its clinical importance, the pathogenesis and progression of DKD remain complex and multifactorial (2).

Dyslipidemia, characterized by elevated triglycerides and reduced high-density lipoprotein cholesterol (HDL-C), is a common metabolic abnormality in DKD patients. Previous studies have demonstrated a strong correlation between dyslipidemia and renal dysfunction in DKD, with persistent dyslipidemia not only exacerbating renal impairment but also increasing cardiovascular mortality risk (3). Among lipid parameters, remnant cholesterol (RC), a novel lipid marker representing the cholesterol content of triglyceride-rich lipoproteins (TRLs), including very low-density lipoproteins (VLDLs), intermediate-density lipoproteins (IDLs), and chylomicron remnants, has garnered increasing attention (4).

Emerging evidence has identified RC as a significant risk factor for atherosclerotic cardiovascular disease (ASCVD), hypertension, and stroke (5–7). Furthermore, RC has been shown to be positively associated with diabetes and serves as an independent predictor of T2D (4, 8). Recent studies have also suggested that RC is an independent risk factor for CKD in patients with prediabetes and T2D (9). However, studies on the relationship between RC and DKD in T2D patients are limited, particularly in the Chinese population. Zhu et al. (9). reported that RC is a risk factor for CKD in prediabetes and T2D patients based on a U.S. population, but their study lacked detailed data on diabetes-related complications and the use of medications such as angiotensin converting enzyme inhibitor (ACEI)/angiotensin II receptor blocker (ARB) or sodium-glucose cotransporter 2 inhibitors (SGLT-2i), which could influence DKD progression. Moreover, their findings suggested a linear relationship between RC and DKD in the NHANES database, but whether this relationship holds true in the Chinese population, with its distinct metabolic and genetic characteristics, remains unclear and warrants further investigation.

This study aims to address these gaps by evaluating the correlation between RC and DKD in a large cohort of Chinese T2D patients. By employing multivariable regression models to adjust for confounding factors, including diabetes-related complications and medication history, this study seeks to provide novel insights into the role of RC as a potential biomarker for early detection and progression monitoring of DKD in Chinese T2D patients.

## Method

### Study population

**Figure 1:**
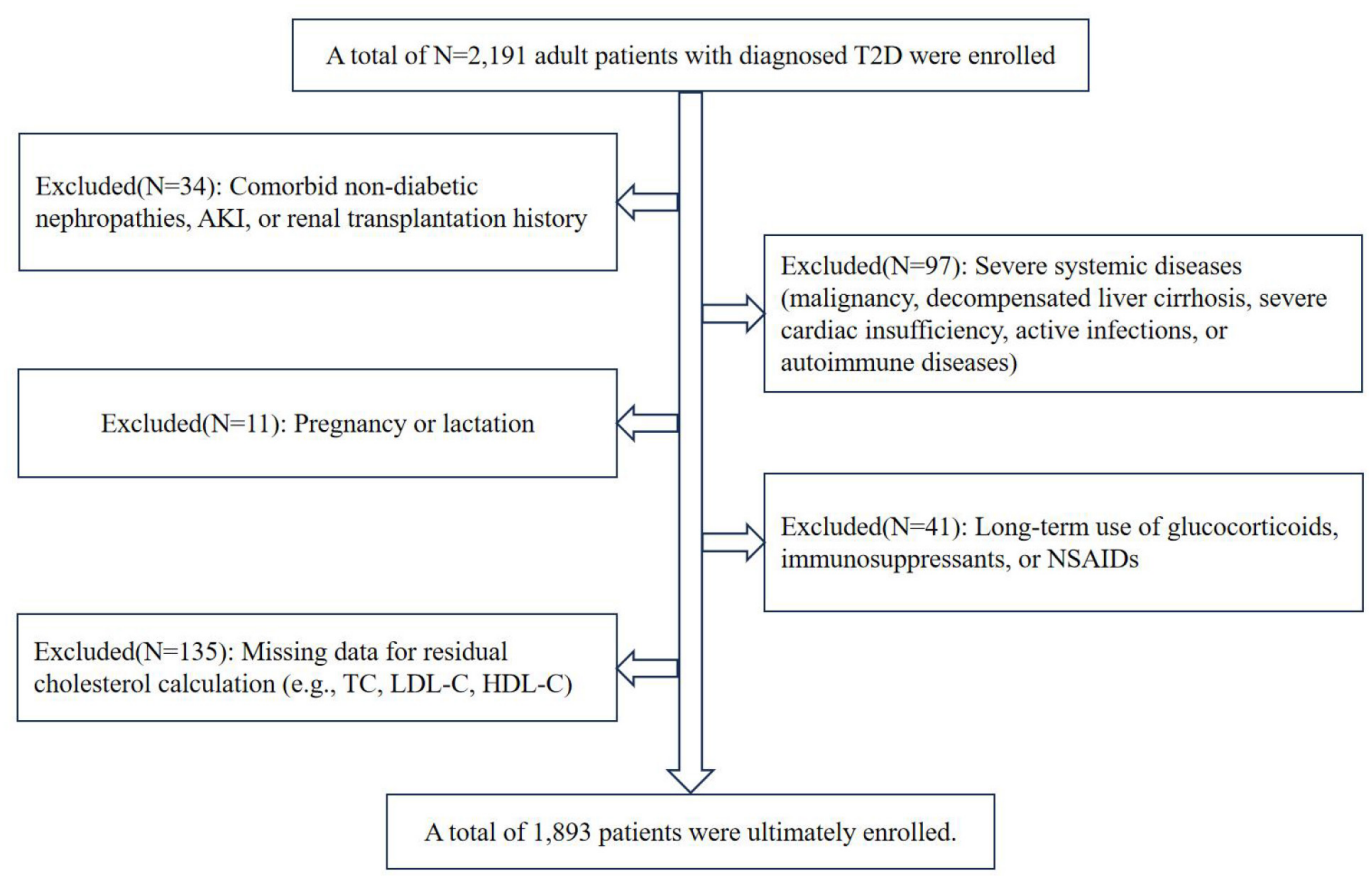
Patient screening process flowchart.

The study selected 1,893 T2D patients hospitalized in Endocrinology Department of the Second Affiliated Hospital of Guangzhou Medical University, Endocrinology Department of the Fourth Affiliated Hospital of Guangzhou Medical University, and Endocrinology Department of the Affiliated Hospital of Guangdong Medical University from 2019 to 2024. The inclusion criteria were adults with newly diagnosed and treated T2D based on the diagnostic criteria recommended by the Chinese Diabetes Society. The exclusion criteria included: (1) Comorbid kidney diseases: non-diabetic nephropathies (e.g., chronic nephritis, glomerulonephritis, polycystic kidney disease); Acute kidney injury (AKI) or renal transplantation history; (2) Severe systemic diseases: Malignancy (currently undergoing chemotherapy or radiotherapy); Decompensated liver cirrhosis; Severe cardiac insufficiency; Active infections or autoimmune diseases (e.g., systemic lupus erythematosus); (3) Pregnancy or lactation; (4) Long-term use of glucocorticoids, immunosuppressants, or non-steroidal anti-inflammatory drugs (NSAIDs); (5) Missing data: Incomplete or absent key variables essential for residual cholesterol (RC) calculation (e.g., total cholesterol, LDL-C, HDL-C). The patient screening flowchart is shown in Figure 1. This retrospective study was approved by the Ethics Committee of The Second Affiliated Hospital of Guangzhou Medical University (Approval number 2020-hs-29), following the Declaration of Helsinki.

### Data collection, measurement and definition

Basic clinical data of the participants were retrospectively collected as follows: gender, age, body mass index (BMI), smoking status, alcohol consumption, medical history, past history including hypertension, coronary atherosclerotic heart disease (CAHD), cerebral infarction (CI) and diabetic complication such as diabetic ketoacidosis (DKA), hyperosmolar hyperglycemic syndrome (HHS), diabetic retinopathy (DR), diabetic peripheral neuropathy (DPN), peripheral vascular disease (PVD), lower extremity arterial occlusive disease (LEAOD) and diabetic foot (DF). BMI was calculated as the ratio of weight in kilograms/height in meters squared. We collected the laboratory tests: fasting blood glucose (FBG), Hemoglobin A1c (HbA1c), fasting C peptide (F-C peptide), cholesterol, triglyceride, low-density lipoprotein cholesterol (LDL-C), low-density lipoprotein cholesterol (HDL-C), serum creatinine (Cr), estimated glomerular filtration rate (eGFR), and urea albumin creatinine ratio (UACR) levels. RC was calculated as TC minus LDL-C minus HDL-C. This estimation method has been recognized as the most common calculation method (10). A systolic blood pressure (SBP) over 140 mmHg or a diastolic blood pressure (DBP) over 90 mmHg, and the use of any anti-hypertensive medication were all considered to be hypertension. Those participants with a UACR ≥ 30 mg/g or an eGFR < 60 ml/min/1.73 m^2^ are considered to have a Diabetic Kidney Disease (DKD), who diagnosed with diabetes based on the above criteria (11).

### Statistical analysis

Continuous variables were expressed as means with standard deviations (SD) for normally distributed data or medians with interquartile ranges (IQR) for non-normally distributed data. Categorical variables were presented as frequencies and percentages. To compare differences between groups, independent *t*-tests or one-way analysis of variance (ANOVA) were applied for normally distributed continuous variables, while the Mann–Whitney *U* test or Kruskal–Wallis test was used for non-normally distributed variables. Chi-square tests were employed for categorical variables. Logistic regression models were constructed to evaluate the association between RC and the risk of DKD. Three models were developed with progressive adjustments: Non-adjusted model included no covariates, Adjust I model adjusted for gender, age and Adjust II model further adjusted for potential confounders selected *a priori* based on clinical relevance and prior literature, including smoking history, diabetic microvascular complications (DR, DPN, DF), macrovascular comorbidities (CAHD, hypertension, CI, PAD), and medication use (ACEI/ARB, SGLT-2i, lipid-lowering agents), because these factors are associated with DKD risk and may also correlate with lipid metabolism. To assess multicollinearity among covariates in the adjusted models, collinearity diagnostics were performed using variance inflation factors (VIFs), and no evidence of problematic multicollinearity was observed. To explore the dose-response relationship between RC and DKD, RC was analyzed both as a continuous variable and in quartiles. Generalized additive models (GAM) with penalized s Adjust II model further adjusted for potential confounders pline functions were used to visualize the potential non-linear relationship between RC and DKD. GAM smoothing was fitted using the mgcv framework with thin plate regression splines (default maximum degrees of freedom = 10), and the smoothing parameter was selected by generalized cross-validation (GCV). A restricted cubic spline (RCS) was additionally used to present the dose–response curve, with 4 knots placed at the 5th, 35th, 65th, and 95th percentiles of RC. Interaction terms were tested using log-likelihood ratio tests to assess heterogeneity across subgroups.

Missingness was quantified for all variables (Supplementary Table S1). Primary analyses were conducted using complete-case data in the final analytic cohort (*N* = 1,893). Because UACR has been routinely implemented in China mainly in the past ~3 years, it was not consistently available earlier; thus, we performed a sensitivity analysis restricted to participants with measured UACR.

All statistical analyses were performed using R software and EmpowerStats (X&Y Solutions, Inc., Boston, MA, USA). A two-tailed *p*-value of < 0.05 was considered statistically significant.

## Results

### Baseline characteristics of participants

A total of 1,893 T2D patients were included in this study, comprising 1,340 without DKD and 553 with DKD. As shown in Table 1, participants with DKD were older and had significantly higher levels of F-C peptide, Cr, UACR, and RC, while HbA1c, FBG, and eGFR were significantly lower compared to those without DKD (*P* < 0.05). Compared with the non-DKD group, the DKD group exhibited significantly higher levels of RC [0.51 (0.36–0.75) mmol/L vs. 0.46 (0.31–0.67) mmol/L, *P* < 0.001]. Additionally, the proportion of newly diagnosed diabetes was higher in the non-DKD group. Regarding complications, the prevalence of DR, PVD, LEAOD, DPN and DF was significantly higher in the DKD group, while DKA and HHS were more frequent in the non-DKD group. The incidence of comorbidities such as CAHD, hypertension, and CI was also significantly higher in the DKD group. Moreover, the use of ACEI/ARB and lipid-lowering drugs was more common in DKD patients, whereas BMI, cholesterol, triglycerides, HDL-C, LDL-C, smoking, drinking, and SGLT-2i use showed no significant differences between the two groups.

**Table 1:** Baseline characteristics of participants according to DKD.

Items	DKD		*P* value
	Without	With	
N	1,340	553	
Age (year)	61 (51–69)	65 (57–72)	< 0.001
Gender: Male(%)	705 (52.61)	298 (53.89)	0.613
BMI (kg/m^2^)	24.10 (21.92–26.75)	24.51 (22.03–26.73)	0.305
HbA1c (%)	9.6 (7.4–11.8)	9.0 (7.3–11.3)	0.009
FBG (mmol/L)	7.35 (5.37–10.19)	6.74 (5.10–9.88)	0.030
F-C peptide (μg/L)	2.03 (1.30–2.87)	2.16 (1.40–3.33)	0.002
Cholesterol (mmol/L)	4.58 (3.84–5.36)	4.56 (3.78–5.46)	0.863
Triglyceride (mmol/L)	1.40 (1.02–2.00)	1.47 (1.02–2.13)	0.157
HDL-C (mmol/L)	1.02 (0.86–1.19)	0.99 (0.84–1.16)	0.052
LDL-C (mmol/L)	3.00 (2.25–3.72)	2.91 (2.18–3.75)	0.753
Cr (μmol/L)	71.00 (59.00–84.00)	82.00 (65.00–114.00)	< 0.001
eGFR (ml/min/1.73 m^2^)	93.26 (76.99–110.31)	78.49 (51.73–100.91)	< 0.001
UACR (mg/gCr)	16.90 (6.95–76.50)	65.40 (20.35–169.40)	< 0.001
RC (mmol/L)	0.46 (0.31–0.67)	0.51 (0.36–0.75)	< 0.001
Newly diagnosed T2D: Yes (%)	473 (35.30)	158 (28.57)	0.005
Drinking: with (%)	109 (8.13)	42 (7.59)	0.694
Smoking: with (%)	269 (20.07)	93 (16.82)	0.101
DKA/HHS: with (%)	138 (10.30)	34 (6.15)	0.004
DR: with (%)	61 (4.55)	89 (16.09)	< 0.001
PVD: with (%)	127 (9.48%)	99 (17.90%)	< 0.001
LEAOD: with (%)	255 (19.03)	181 (32.73)	< 0.001
DPN: with (%)	465 (34.70)	271 (49.01)	< 0.001
DF: with (%)	28 (2.09)	22 (3.98)	0.020
CAHD: with (%)	135 (10.07)	111 (20.07)	< 0.001
Hypertension: with (%)	590 (44.03)	392 (70.89)	< 0.001
CI: with (%)	210 (15.67)	154 (27.85)	< 0.001
ACEI/ARB: with (%)	424 (31.64)	259 (46.84)	< 0.001
SGLT-2i: with (%)	298 (22.24)	138 (24.95)	0.202
Lipid-lowering drug: with (%)	550 (41.04)	281 (50.81)	< 0.001

BMI, body mass index; HbA1c, hemoglobin A1c; FBG, fasting blood glucose; F-C peptide, fasting C peptide; HDL-C, high-density lipoprotein cholesterol; LDL-C, low-density lipoprotein cholesterol; Cr, creatinine; eGFR, estimated glomerular filtration rate; UACR, urea albumin creatinine ratio; RC, remnant cholesterol; DKA, diabetic ketoacidosis; HHS, hyperosmolar hyperglycemic syndrome; DR, diabetic retinopathy; PVD, peripheral vascular disease; LEAOD, lower extremity arterial occlusive disease; DPN, diabetic peripheral neuropathy; DF, diabetic foot; CAHD, coronary atherosclerotic heart disease; CI, cerebral infarction; ACEI, angiotensin converting-enzyme inhibitor; ARB, angiotensin II receptor blocker; SGLT-2i, sodium-glucose cotransporter protein-2 inhibitors.

### Association analysis between RC and DKD

Logistic regression models were constructed to evaluate the association between RC and DKD. In the non-adjusted model, RC as a continuous variable was significantly associated with an increased risk of DKD (OR= 1.39, 95% CI = 1.10–1.75). This association remained significant in adjusted I (OR = 1.52, 95% CI = 1.20–1.93) and adjusted II models (OR =1.43, 95% CI =1.10–1.86) (Table 2).

**Table 2:** Correlation of RC and DKD in T2D patients using multiple models.

Exposure	Non-adjusted	Adjust I	Adjust II
Continuous RC	1.39 (1.10, 1.75)	1.52 (1.20, 1.93)	1.43 (1.10, 1.86)
RC quartile			
Q1 ( ≤ 0.32)	1.0	1.0	1.0
Q2 (0.330, 0.470)	1.42 (1.06, 1.91)	1.36 (1.01, 1.82)	1.22 (0.89, 1.69)
Q3 (0.480, 0.690)	1.54 (1.15, 2.07)	1.53 (1.14, 2.06)	1.42 (1.03, 1.96)
Q4 (≥0.700)	1.85 (1.38, 2.47)	1.97 (1.47, 2.65)	1.77 (1.28, 2.45)
*P* for trend	< 0.001	< 0.001	< 0.001

OR, odds ratio; 95%CI, 95% confidence interval.

Non-adjusted model: adjust for none Adjust I model: adjusted for gender, age Adjust II model: adjusted for gender, age, smoking history, DR, PAD, DPN, DF, CAHD, hypertension, CI, ACEI/ARB, SGLT-2i and lipid-lowering agents.

When RC was treated as a categorical variable, the highest quartile of RC was associated with a significantly higher DKD risk compared to the lowest quartile (OR = 1.85, 95% CI = 1.38–2.47, *P* < 0.001). Adjusted II models showed a similar trend, with ORs for the 2nd, 3rd, and 4th quartiles of 1.22 (95% CI = 0.89–1.69, *P* = 0.21), 1.42 (95% CI = 1.03–1.96, *P* = 0.03), and 1.77 (95% CI = 1.28–2.45, *P* = 0.001), respectively (Table 2). Furthermore, a significant trend was observed across quartiles in the adjusted models (*P* for trend < 0.001), indicating a dose-response relationship between RC levels and DKD risk. These findings suggest that higher RC levels are consistently associated with an increased risk of DKD, even after adjusting for potential confounders.

**Figure 2:**
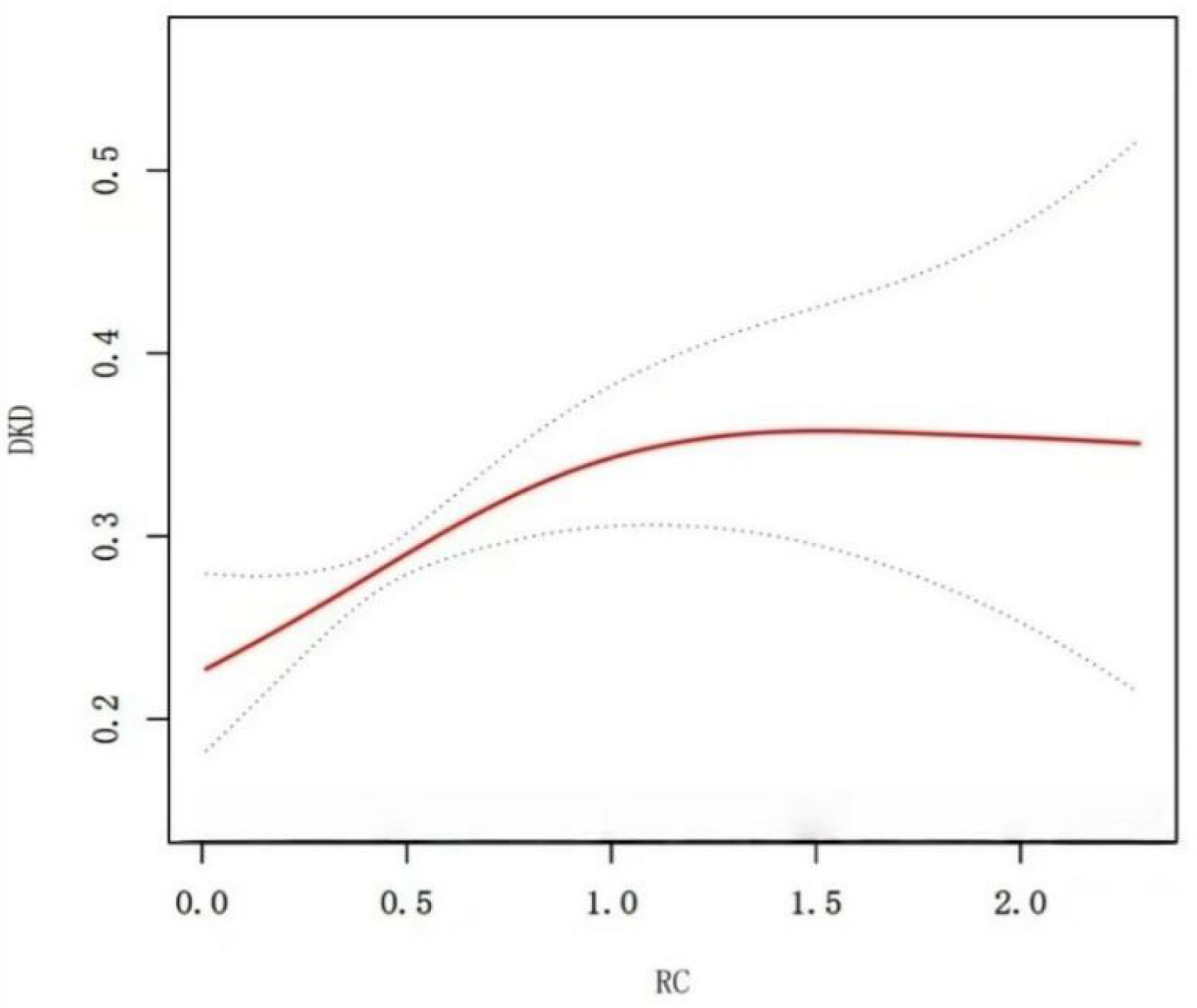
Restricted cubic spline (RCS) plots to demonstrate the level of RC in T2DM patients in relation to the occurrence of DKD. The red solid line indicates the smooth curve fit between the variables. The blue bands represent the 95% of confidence interval from the fit. The covariates that were adjusted to the model were the same as described above.

The RCS model revealed a significant linear increase in DKD risk with RC levels below 0.96, indicating a steady rise in risk of DKD as RC levels increased. However, when RC levels exceeded 0.96, the DKD risk exhibited a non-linear pattern, with the risk increasing at a slower rate compared to the linear phase (Figure 2 and Table 3).

**Table 3:** Threshold effects analysis of RC and DKD using liner regression models.

RC	DKD (β, 95%CI, *P* value)
Total	
The standard linear model	1.43 (1.10, 1.86) 0.007
Model	
Infection point (K)	0.96
RC < 0.96	2.25 (1.37, 3.68) 0.001
RC >0.96	0.86 (0.49, 1.49) 0.588
*P* for Log-likelihood ratio	0.036

Th model was adjusted for gender, age, smoking history, DR, PAD, DPN, DF, CAHD, hypertension, CI, ACEI/ARB, SGLT-2i and lipid-lowering drug.

### Subgroup and sensitivity analyses

**Figure 3:**
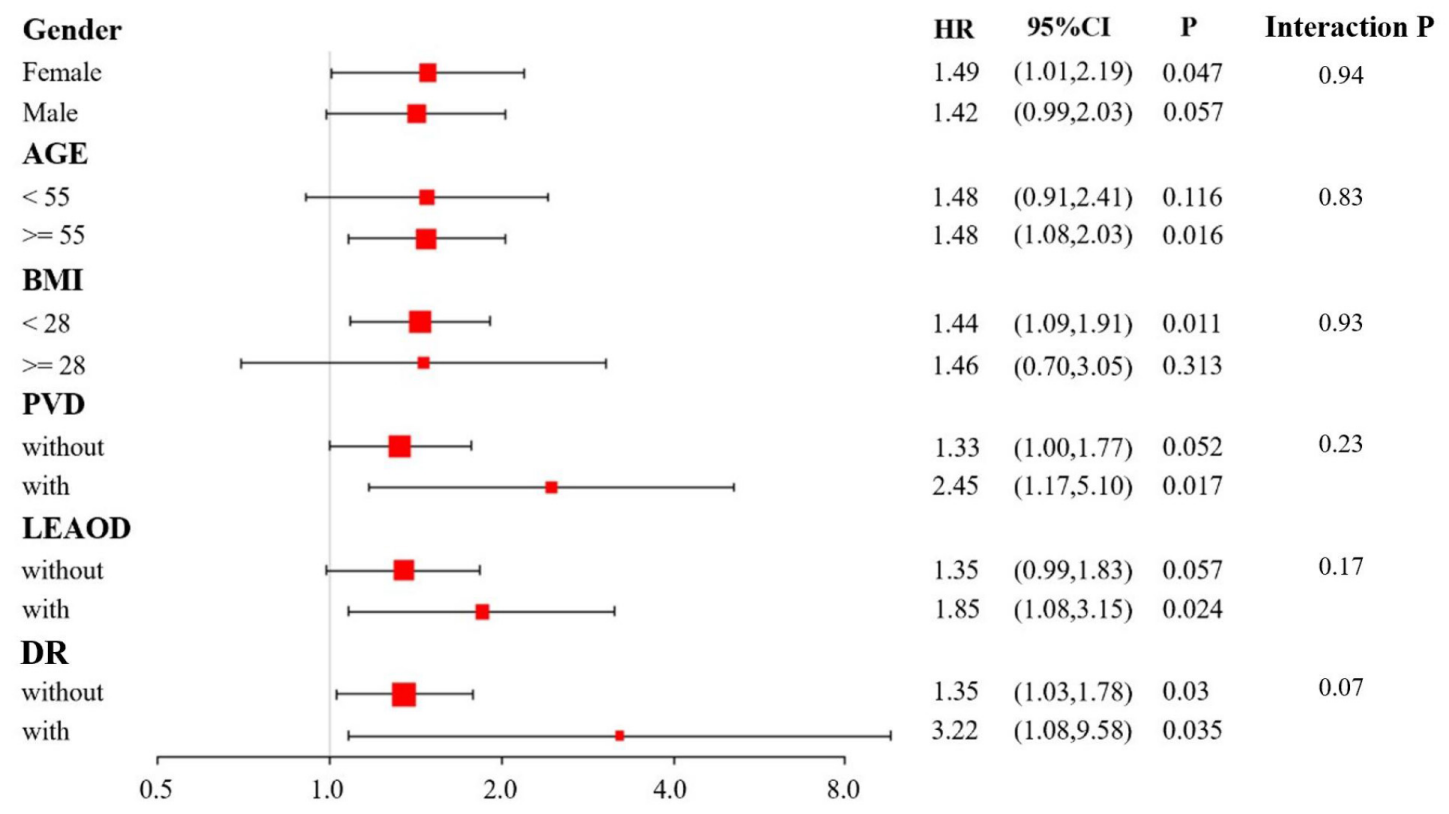
The association between RC and the occurrence of DKD by subgroup analysis in T2D patients. The RC was analyzed using continuous variables, and interaction tests were also conducted to derive the interaction *P*-value. The black line segments represent the 95% CI for each group, and the ends of the lines represent the upper and lower 95% CI limits. The black diamond reflects the midpoint of the line segment and illustrates the effect value.

Subgroup analysis showed no significant interactions between RC and DKD risk across subgroups stratified by gender, age, BMI, RA, PVD, and LEAOD (all *P* for interaction > 0.05), indicating the robustness of the association (Figure 3). Notably, several subgroup-specific associations reached statistical significance (*P* < 0.05), which reflects within-subgroup effects rather than evidence of effect modification.

In sensitivity analyses restricted to participants with measured UACR, the association between RC and DKD remained consistent with the main analysis (Supplementary Table S2).

## Discussion

This cross-sectional study was aimed to explore the underlying relationship of RC to DKD in Chinese patients with T2D. Our findings demonstrate that in T2D patients with DKD the RC level was significantly higher that those without DKD [median (IQR): 0.51 (0.36–0.75) vs. 0.46 (0.31–0.67) mmol/L, *P* < 0.001]. Multivariable logistic regression revealed a 43% increased DKD risk per 0.1 mmol/L RC increment (adjusted OR = 1.43, 95%CI = 1.10–1.86). When analyzed as quartiles, participants in the highest RC quartile (Q4) demonstrated 1.77-fold higher DKD risk compared to the lowest quartile (Q1) (95%CI = 1.28–2.45, *P* = 0.001), with significant linear trend across quartiles (*P* < 0.001). Furthermore, RCS plots demonstrated a biphasic relationship: below 0.96 mmol/L, risk increased linearly with RC (β = 2.25 per 0.1 mmol/L, *P* = 0.001), transitioning to a plateau (β = 0.86, *P* = 0.588) above this threshold, suggesting lipid-mediated renal injury pathways may reach saturation. The biphasic relationship in our study may was similar to previous study reported a steep-rise–plateau pattern between RC and DKD risk (12). Dai et al. (12) speculated that lower to moderate RC levels may markedly amplify endothelial dysfunction, and lipid deposition, triggering inflammatory and fibrotic pathways to accelerate renal injury. However, once these pathogenic pathways are maximally activated, further higher RC level may confer limited additional risk, contributing to a saturation-like plateau. Additionally, the subgroup analysis confirmed the correlation between RC and the risk of DKD in T2D patients. These results suggested that elevated RC levels represent a potential risk factor for DKD in T2D patients.

In recent years, RC has garnered significant attention as a novel atherosclerotic lipid indicator. Its pathophysiological significance stems from its heterogeneous characteristics—RC represents cholesterol content in a group of atherogenic remnant lipoprotein particles (including very-low-density lipoprotein remnants and chylomicron remnants) with distinct densities, volumes, and apolipoprotein compositions (13, 14). Notably, RC's metabolic disruption extends beyond the cardiovascular system. Large-scale population studies reveal its significant association with the risk of T2D development (15), where elevated baseline RC levels can increase diabetes onset risk (16). Furthermore, the association between RC and macrovascular complications in diabetes has been validated in multiple studies (17, 18). While RC has been well-established as a pathological factor in macrovascular complications of diabetes (17, 18), its role in microvascular complications remains under-explored. Zhu et al. (9) demonstrated RC's association with CKD risk in prediabetes and T2D populations but failed to distinguish DKD from non-DKD. Notably, their U.S.-based cohort lacks documentation of ACEI/ARB and SGLT-2i usage—critical medications influencing DKD progression. Cross-sectional studies associate RC with DR in T2D (19), while Jansson Sigfrids et al. (20) extended these findings to DR and DKD in type 1 diabetes mellitus (T1D) populations, suggesting RC's renal effects may transcend diabetes subtypes. Although Wu et al. (21). Firstly reported the RC-DKD association in T2D, their Martin-Hopkins equation for RC calculation (requiring dynamic adjustment based on triglycerides and non-HDL cholesterol) has proven clinically impractical. Furthermore, their model omitted adjustments for nephroprotective agents. To address these gaps, our study adopted the consensus formula (RC = TC – LDL-C – HDL-C) (4, 10), which balances scientific validity with clinical utility, and incorporated ACEI/ARB/SGLT-2i usage into multivariate models. Additionally, we established a dose-response RC-DKD relationship in Chinese T2D participants through RCS analysis.

This study confirmed through logistic regression models that the positive association between RC and DKD in T2D patients remains statistically significant, regardless of whether RC was treated as a continuous or categorical variable. The association persisted even after controlling for confounding factors including gender, age, smoking history, DR, PAD, DPN, DF, CAHD, hypertension, CI, and the use of ACEI/ARBs, SGLT-2 inhibitors, and lipid-lowering agents. Notably, while prior studies inadequately controlled for the potential influence of ACEI/ARBs, SGLT-2i, and lipid-lowering agents on DKD progression or RC levels, this study addressed these methodological gaps through rigorous adjustments. RCS modeling further revealed a biphasic dose-response relationship between RC and DKD risk: a linear increase in DKD risk was observed with rising RC levels at RC concentrations < 0.96 mmol/L, whereas the risk trajectory transitioned to a non-linear pattern when RC levels exceeded this threshold. This non-linear relationship suggests potential biological saturation of RC-mediated renal injury mechanisms beyond specific concentration thresholds. In subgroup analyses, the positive association between RC and DKD was directionally consistent across strata of sex, age, BMI, RA, PVD, and LEAOD, and no statistically significant effect modification was detected (all *P* for interaction > 0.05). Although several strata showed *P* < 0.05 for the within-subgroup association, these *P* values reflect statistical evidence within that subgroup and do not imply heterogeneity of effects between subgroups. The apparent differences in significance across strata are likely related to sample size and event distribution, and multiple subgroup comparisons may also yield nominally significant findings. Overall, the subgroup results support the robustness of the RC–DKD association rather than identifying a specific high-risk subgroup with a clearly different effect size. Conventional lipid parameters (e.g., triglycerides, HDL-C) have been previously validated in epidemiological and genetic studies as correlates of DKD (22–24). Our findings extend this evidence by identifying RC as an independent novel lipid biomarker associated with DKD, thereby augmenting the dyslipidemia theory of diabetic nephropathy. However, the precise molecular mechanisms underlying RC-induced renal injury remain unclear. Current hypotheses focus on three primary mechanisms: (1) the Low-Grade Inflammatory Pathway, where remnant lipoproteins activate nuclear factor κB (NF-κB) signaling through Toll-like receptor 4 (TLR4), promoting inflammatory cytokine secretion in glomerular mesangial cells (25). Inflammation is one of dominant factors that promote the progression of DKD (26). Genome-wide transcriptome analysis studies reported that inflammatory signaling pathway including NF-κB/TLR4 are closely associated with DKD development (27). Previous study reported that TLR4/NF-κB signaling could induce pyroptosis in tubular cells in DKD (28). Previous evidence has identified that RC was associated that NF-κB signaling (29, 30). (2) mTOR is a central regulator of metabolism and cell growth in response to nutrient signaling (31, 32). Activation of mTOR signaling is a key pathological node in DKD progression, driving renal injury through multiple pathways (33, 34). RC is essentially the cholesterol in the remnants of TRL. Excessive nutrients or energy can lead to dyslipidemia including the increase of RC level. And RC increase also induced energy metabolism disorder. Therefore, the mTOR signaling pathway potentially may serve as a mediator linking RC to the occurrence and development of DKD. However, the specific molecular mechanism of the effect of RC on DKD still requires further investigation. (3) The insulin resistance-mediated pathway, in which elevated RC levels create a lipotoxic microenvironment that exacerbates insulin signaling dysfunction in renal tubular epithelial cells. Higher RC level was associated with increased prevalence of IR and T2D (35). IR drives the progression of DKD through multiple pathways including metabolic disorder, inflammation and oxidative stress. IR also contributed to increase the free fatty acid (FFA) levels, lipid deposition in the kidney. We speculated that elevated RC levels create a lipotoxic microenvironment for kidney mediated by insulin resistance. However, the specific mechanism still awaits further researches to validate (36). Future mechanistic studies utilizing animal models with temporal and spatial resolution, combined with cellular/molecular approaches, will be essential to validate these mechanistic hypotheses and explore the therapeutic potential of RC-targeted interventions for diabetic nephropathy.

This study still has certain limitations. First of all, because of its cross-sectional observation and inadequate sample sizes, it was not feasible to establish a clear causal link between RC and DKD. Secondly, since RC was calculated by the formula and not directly measured, the results are subject to error which would affect its accuracy. However, in practice, it is relatively simple and easy to calculate RC in large-scale population studies. Finally, we didn't specifically categorize lipid-lowering drugs when discussing whether they could be a biasing factor for RC because of the insufficient sample size. Future studies should collect larger sample sizes and conduct prospective studies to further investigate the relationship between RC and DKD.

In conclusion, this study establishes RC as an independent risk indicator of diabetic kidney disease in Chinese T2D patients, revealing a novel biphasic threshold effect with distinct linear and nonlinear risk trajectories. These findings position RC as both a pathophysiological marker for DKD prevention.

## Data Availability

The raw data supporting the conclusions of this article will be made available by the authors, without undue reservation.

## References

[1] Rayego-Mateos S Rodrigues-Diez RR Fernandez-Fernandez B Mora-Fernández C Marchant V Donate-Correa J . Targeting inflammation to treat diabetic kidney disease: the road to 2030. Kidney Int. (2023) 103:282–96. doi: 10.1016/j.kint.2022.10.030 36470394

[2] Tuttle KR Agarwal R Alpers CE Bakris GL Brosius FC Kolkhof P . Molecular mechanisms and therapeutic targets for diabetic kidney disease. Kidney Int. (2022) 102:248–60. doi: 10.1016/j.kint.2022.05.012 35661785

[3] Su W Cao R He YC Guan YF Ruan XZ. Crosstalk of hyperglycemia and dyslipidemia in diabetic kidney disease. Kidney Dis. (2017) 3:171–80. doi: 10.1159/000479874 29344511 PMC5757547

[4] Huh JH Roh E Lee SJ Ihm SH Han KD Kang JG. Remnant cholesterol is an independent predictor of type 2 diabetes: a nationwide population-based cohort study. Diabetes Care. (2023) 46:305–12. doi: 10.2337/dc22-1550 36469354

[5] Burnett JR Hooper AJ Hegele RA. Remnant cholesterol and atherosclerotic cardiovascular disease risk. J Am Coll Cardiol. (2020) 76:2736–9. doi: 10.1016/j.jacc.2020.10.029 33272367

[6] Guo DC Gao JW Wang X Chen ZT Gao QY Chen YX . Remnant cholesterol and risk of incident hypertension: a population-based prospective cohort study. Hypertens Res. (2024) 47:1157–66. doi: 10.1038/s41440-023-01558-7 38212367

[7] Li W Huang Z Fang W Wang X Cai Z Chen G . Remnant cholesterol variability and incident ischemic stroke in the general population. Stroke. (2022) 53:1934–41. doi: 10.1161/STROKEAHA.121.037756 35543132

[8] Li B Liu Y Zhou X Chen L Yan L Tang X . Remnant cholesterol is more positively related to diabetes, prediabetes, and insulin resistance than conventional lipid parameters and lipid ratios: a multicenter, large sample survey. J Diabetes. (2024) 16:e13592. doi: 10.1111/1753-0407.13592 39136535 PMC11320755

[9] Zhu W Liu Q Liu F Jiao C Zhang L Xie H. High remnant cholesterol as a risk factor for developing chronic kidney disease in patients with prediabetes and type 2 diabetes: a cross-sectional study of a US population. Acta Diabetol. (2024) 61:735–43. doi: 10.1007/s00592-024-02249-6 38436703 PMC11101368

[10] Tian Y Wu Y Qi M Song L Chen B Wang C . Associations of remnant cholesterol with cardiovascular and cancer mortality in a nationwide cohort. Science bulletin. (2024) 69:526–34. doi: 10.1016/j.scib.2023.12.035 38155000

[11] KDIGO 2020 Clinical practice guideline for diabetes management in chronic kidney disease. Kidney Int. (2020) 98:S1–115. doi: 10.1016/j.kint.2020.06.019 32998798

[12] Dai Y Pan Q Yu Y Ma Y Chen G Wang H. Remnant cholesterol for diabetic kidney disease risk stratification in type 2 diabetes: a machine learning-based prevention tool. Front Nutr. (2025) 12:1697943. doi: 10.3389/fnut.2025.1697943 41306673 PMC12643847

[13] Castañer O Pintó X Subirana I Amor AJ Ros E Hernáez Á . Remnant cholesterol, not LDL cholesterol, is associated with incident cardiovascular disease. J Am Coll Cardiol. (2020) 76:2712–24. doi: 10.1016/j.jacc.2020.10.008 33272365

[14] Varbo A Benn M Tybjærg-Hansen A Jørgensen AB Frikke-Schmidt R Nordestgaard BG. Remnant cholesterol as a causal risk factor for ischemic heart disease. J Am Coll Cardiol. (2013) 61:427–36. doi: 10.1016/j.jacc.2012.08.1026 23265341

[15] American Diabetes Association Professional Practice Committee. Classification and diagnosis of diabetes: standards of medical care in diabetes-2022. Diabetes Care. (2022) 45:S17–38. doi: 10.2337/dc22-S002 34964875

[16] Xie G Zhong Y Yang S Zou Y. Remnant cholesterol is an independent predictor of new-onset diabetes: a single-center cohort study. Diabetes Metab Syndr Obes Targets Ther. (2021) 14:4735–45. doi: 10.2147/DMSO.S341285 34887671 PMC8652915

[17] Cao YX Zhang HW Jin JL Liu HH Zhang Y Gao Y . The longitudinal association of remnant cholesterol with cardiovascular outcomes in patients with diabetes and pre-diabetes. Cardiovasc Diabetol. (2020) 19:104. doi: 10.1186/s12933-020-01076-7 32631321 PMC7339517

[18] Fu L Tai S Sun J Zhang N Zhou Y Xing Z . Remnant cholesterol and its visit-to-visit variability predict cardiovascular outcomes in patients with type 2 diabetes: findings from the ACCORD cohort. Diabetes Care. (2022) 45:2136–43. doi: 10.2337/dc21-2511 35834242 PMC9472497

[19] Chen S Xu Y Chen B Lin S Lu L Cheng M . Remnant cholesterol is correlated with retinal vascular morphology and diabetic retinopathy in type 2 diabetes mellitus: a cross-sectional study. Lipids Health Dis. (2024) 23:75. doi: 10.1186/s12944-024-02064-6 38468242 PMC10926603

[20] Jansson Sigfrids F Dahlström EH Forsblom C Sandholm N Harjutsalo V Taskinen MR . Remnant cholesterol predicts progression of diabetic nephropathy and retinopathy in type 1 diabetes. J Intern Med. (2021) 290:632–45. doi: 10.1111/joim.13298 33964025

[21] Wu Z Yu S Zhu Q Li Z Zhang H Kang X . Association of baseline and cumulative remnant cholesterol with incidence of diabetic nephropathy: a longitudinal cohort study. Diabetes Res Clin Pract. (2022) 191:110079. doi: 10.1016/j.diabres.2022.110079 36099974

[22] Sacks FM Hermans MP Fioretto P Valensi P Davis T Horton E . Association between plasma triglycerides and high-density lipoprotein cholesterol and microvascular kidney disease and retinopathy in type 2 diabetes mellitus: a global case-control study in 13 countries. Circulation. (2014) 129:999–1008. doi: 10.1161/CIRCULATIONAHA.113.002529 24352521

[23] Russo GT De Cosmo S Viazzi F Pacilli A Ceriello A Genovese S . Plasma triglycerides and HDL-C levels predict the development of diabetic kidney disease in subjects with type 2 diabetes: the AMD annals initiative. Diabetes Care. (2016) 39:2278–87. doi: 10.2337/dc16-1246 27703024

[24] Lin J Hu FB Mantzoros C Curhan GC. Lipid and inflammatory biomarkers and kidney function decline in type 2 diabetes. Diabetologia. (2010) 53:263–7. doi: 10.1007/s00125-009-1597-z 19921505 PMC2809803

[25] Doi H Kugiyama K Oka H Sugiyama S Ogata N Koide SI . Remnant lipoproteins induce proatherothrombogenic molecules in endothelial cells through a redox-sensitive mechanism. Circulation. (2000) 102:670–6. doi: 10.1161/01.CIR.102.6.670 10931808

[26] Jung SW Moon JY. The role of inflammation in diabetic kidney disease. Korean J Intern Med. (2021) 36:753–66. doi: 10.3904/kjim.2021.174 34237822 PMC8273831

[27] Sandholm N Cole JB Nair V Sheng X Liu H Ahlqvist E . Genome-wide meta-analysis and omics integration identifies novel genes associated with diabetic kidney disease. Diabetologia. (2022) 65:1495–509. doi: 10.1007/s00125-022-05735-0 35763030 PMC9345823

[28] Wang Y Zhu X Yuan S Wen S Liu X Wang C . TLR4/NF-κB signaling induces GSDMD-related Pyroptosis in tubular cells in diabetic kidney disease. Front Endocrinol. (2019) 10:603. doi: 10.3389/fendo.2019.00603 31608008 PMC6761221

[29] Park SY Lee JH Kim YK Kim CD Rhim BY Lee WS . Cilostazol prevents remnant lipoprotein particle-induced monocyte adhesion to endothelial cells by suppression of adhesion molecules and monocyte chemoattractant protein-1 expression via lectin-like receptor for oxidized low-density lipoprotein receptor activation. J Pharmacol Exp Ther. (2005) 312:1241–8. doi: 10.1124/jpet.104.077826 15525793

[30] Ding Y Wang L Sun J Shi Y Li G Luan X . Remnant cholesterol and dyslipidemia are risk factors for Guillain–Barré syndrome and severe Guillain–Barré syndrome by promoting monocyte activation. Front Immunol. (2022) 13:946825. doi: 10.3389/fimmu.2022.946825 35911688 PMC9326451

[31] Zhao T Fan J Abu-Zaid A Burley SK Zheng XFS. Nuclear mTOR signaling orchestrates transcriptional programs underlying cellular growth and metabolism. Cells. (2024) 13:781. doi: 10.3390/cells13090781 38727317 PMC11083943

[32] Fan J Zhang X Zhang J Zhao T Burley SK Zheng XFS. PDX1 phosphorylation at S61 by mTORC1 links nutrient signaling to β cell function and metabolic disease. Cell Rep. (2026) 45:116811. doi: 10.1016/j.celrep.2025.116811 41528843 PMC12949489

[33] Dong R Zhang X Liu Y Zhao T Sun Z Liu P . Rutin alleviates EndMT by restoring autophagy through inhibiting HDAC1 via PI3K/AKT/mTOR pathway in diabetic kidney disease. Phytomedicine. (2023) 112:154700. doi: 10.1016/j.phymed.2023.154700 36774842

[34] Li T Chen H Guo Y Huang M Liu P Aikemu A . Nuciferine restores autophagy via the PI3K-AKT-mTOR pathway to alleviate renal fibrosis in diabetic kidney disease. J Agric Food Chem. (2025) 73:5223–35. doi: 10.1021/acs.jafc.4c08844 39989251

[35] Li Y Zeng Q Peng D Hu P Luo J Zheng K . Association of remnant cholesterol with insulin resistance and type 2 diabetes: mediation analyses from NHANES 1999-2020. Lipids Health Dis. (2024) 23:404. doi: 10.1186/s12944-024-02393-6 39695677 PMC11653793

[36] Kume S Uzu T Araki S Sugimoto T Isshiki K Chin-Kanasaki M . Role of altered renal lipid metabolism in the development of renal injury induced by a high-fat diet. J Am Soc Nephrol JASN. (2007) 18:2715–23. doi: 10.1681/ASN.2007010089 17855643

