# Supplementary material for: Exploring the relationship between remnant cholesterol and diabetic kidney disease in Chinese type 2 diabetes patients
Source: Front Nutr. 2026 Mar 30;13:1793007. doi: 10.3389/fnut.2026.1793007 (PMC13070960; doi:10.3389/fnut.2026.1793007)
Supplement: Supplementary file 1 [file Table_1.docx]

**Supplementary Table S1. Missing data pattern and handling strategy**

| Variable | Missing, (%) | Handling in analysis |
| --- | --- | --- |
| Age | 0.05 | Complete-case in regression (missingness low) |
| Gender | 0.00 | Complete-case in descriptive/regression analyses |
| BMI | 0.00 | Complete-case in descriptive/regression analyses |
| HbA1c | 5.12 | Complete-case in descriptive/regression analyses |
| FBG | 2.06 | Complete-case in descriptive/regression analyses |
| F- C-peptide | 3.70 | Complete-case in descriptive/regression analyses |
| Cholesterol | 0.00 | - |
| Triglyceride | 0.00 | Complete-case in descriptive/regression analyses |
| HDL-C | 0.00 | - |
| LDL-C | 0.00 | - |
| Cr | 2.75 | Complete-case in descriptive/regression analyses |
| eGFR | 2.85 | Complete-case in descriptive/regression analyses |
| UACR | 49.87 | High missingness; sensitivity analysis restricted to measured UACR |
| RC | 0.00 | - |
| Newly diagnosed T2D | 0.00 | Complete-case in descriptive/regression analyses |
| Smoking | 0.00 | Complete-case in descriptive/regression analyses |
| Drinking | 0.00 | Complete-case in descriptive/regression analyses |
| DKA/HHS | 0.00 | Complete-case in descriptive/regression analyses |
| DR | 0.00 | Complete-case in descriptive/regression analyses |
| PVD | 0.00 | Complete-case in descriptive/regression analyses |
| LEAOD | 0.00 | Complete-case in descriptive/regression analyses |
| DPN | 0.00 | Complete-case in descriptive/regression analyses |
| DF | 0.00 | Complete-case in descriptive/regression analyses |
| CAHD | 0.00 | Complete-case in descriptive/regression analyses |
| Hypertension | 0.00 | Complete-case in descriptive/regression analyses |
| CI | 0.00 | Complete-case in descriptive/regression analyses |
| ACEI/ARB | 1.27 | Complete-case in regression (missingness low) |
| SGLT-2i | 0.00 | Complete-case in descriptive/regression analyses |
| Lipid-lowering drug use | 0.11 | Complete-case in regression (missingness low) |

BMI: body mass index; HbA1c: Hemoglobin A1c; FBG: fasting blood glucose; F-C peptide : fasting C peptide; HDL-C: high-density lipoprotein cholesterol; LDL-C: low-density lipoprotein cholesterol; Cr: creatinine; eGFR: estimated glomerular filtration rate; UACR: urea albumin creatinine ratio; RC: remnant cholesterol; DKA: diabetic ketoacidosis; HHS: hyperosmolar hyperglycemic syndrome; DR: diabetic retinopathy; PVD: peripheral vascular disease; LEAOD: lower extremity arterial occlusive disease; DPN: diabetic peripheral neuropathy; DF: diabetic foot; CAHD: coronary atherosclerotic heart disease; CI: cerebral infarction; ACEI: angiotensin converting-enzyme inhibitor; ARB: angiotensin II receptor blocker; SGLT-2i: Sodium-glucose cotransporter protein-2 inhibitors.

**Supplementary Table S2. Sensitivity analysis restricted to participants with measured UACR**(N=949)

| Exposure | Non-adjusted | Adjust I | Adjust II |
| --- | --- | --- | --- |
| Continuous RC | 1.45 (1.13, 1.87) | 1.66 (1.28, 2.17) | 1.55 (1.17, 2.05) |
| RC quartile |  |  |  |
| Q1 (≤0.32) | 1.0 | 1.0 | 1.0 |
| Q2 (0.330, 0.470) | 1.75 (1.12, 2.74) | 1.61 (1.02, 2.54) | 1.53 (0.95, 2.49) |
| Q3 (0.480, 0.690) | 1.94 (1.24, 3.04) | 1.83 (1.16, 2.89) | 1.63 (1.00, 2.64) |
| Q4 (≥0.700) | 2.75 (1.78, 4.24) | 3.02 (1.94, 4.70) | 2.69 (1.69, 4.29) |
| P for trend | <0.001 | <0.001 | <0.001 |

OR: odds ratio; 95%CI: 95% confidence interval

Non-adjusted model: adjusted for none

Adjust I model: adjusted for gender and age

Adjust II model: adjusted for gender, age, smoking history, DR, PAD, DPN, DF, CAHD, hypertension, CI, ACEI/ARB, SGLT-2i and lipid-lowering agents
